# Knowledge and misconceptions about HIV counseling and testing (HCT) among the post-conflict youths of Gulu, Northern Uganda. A prospective study design

**Published:** 2012-06-14

**Authors:** David Lagoro Kitara, Charles Amone, Christopher Okello

**Affiliations:** 1 Gulu University, Gulu, Uganda

**Keywords:** Knowledge, youths, HIV counseling and testing (HCT), post-conflict, Uganda

## Abstract

**Background:**

Uganda has been reported as the most successful country in Africa in reducing the prevalence of HIV/AIDS from 18% to 6.4% over the last two decades. There is evidence to suggest that despite a significant decline between 1992 and 2002, HIV prevalence has stagnated over the last 5-9 years at between 6.1 and 6.5% and it is rising in some parts of the country such as Gulu. This rise are thought to be due to the high levels of stigma and superstition preventing HIV counseling and testing (HCT). WHO reports in 2009 showed that only 20% of Uganda's populations knew their HIV sero-status. This study was designed to find out the knowledge, misconceptions, attitude and practices of youths of Gulu about HCT.

**Methods:**

A cross-sectional study was conducted in Gulu, Pece among the youths 15 to 35 years. An in-depth interview using a questionnaire was administered to youths of Commercial Road Sub-ward. Informed consent and ethical approval was obtained and 86 respondents were interviewed.

**Results:**

Ninety three percent of respondents had knowledge about HCT and 97.7% were able to mention two or more of its benefits. Most (88.4%) agreed on public disclosure of their HIV status and 84.9% would encourage others to undertake it. Only 36.1% of respondents had undertaken HCT while the rest had not undertaken it due to fear of stigmatization.

**Conclusion:**

There is adequate knowledge, good attitude but poor practice and misconceptions to HCT. The young adults in Gulu should be supported in a special program to enable them undertake HCT and access other services for HIV/AIDS prevention.

## Background

Counseling and testing services for HIV (HCT) have long been a component of HIV prevention and care programs in developed countries and proved to be a cost-effective way of reducing the risky behaviours and of leading patients to other services [1]. When HIV/AIDS blood testing became available in Uganda at the middle of 1985, it was immediately put into use all over the country in over 90% of facilities that collected blood for blood transfusion [2]. The wide spread acceptance and the use of blood testing for HIV/AIDS helped reduce the spread of the virus [2, 3]. This was achieved through adequate counseling to explain the meaning of a positive test result, provision of psychosocial support to ease the shock of a positive test result and the fact that many people accepted the result and pronounced their positive lives in public without the fear of stigmatization [3]. This created the biggest impact on the fight against HIV/AIDS in Uganda and its current prevalence which declined from 29% in the 80s to less than 10% in the year 2000 [3]. HIV counseling and testing is considered a pivotal service and a vital entry point in the management of HIV/AIDS; providing a continuum for HIV prevention and provision of care, treatment and support services [4, 5]. HCT helps people to cope with their personal stress and make decisions related to HIV [5]. HCT is the best and most objective way to diagnose HIV infection and prevent the spread of the virus; as opposed to testing without counseling and emphasis on the behaviour change [4, 6]. There are various categories of HCT and these include; voluntary counseling and testing (VCT) [7], Routine Counseling and Testing (RCT) [7, 8] and home-based counseling and testing (HBCT) [7]. All these efforts were to make HIV screening services accessible to more people, especially in rural areas where there were neither modern laboratories nor electricity to run standard HIV tests [7, 8]. The HCT services are now available in all districts in Uganda but the uptake is still low, though it is reported that the numbers are slowly increasing [7]. A systematic review of data from Kenya, Tanzania and Trinidad documented a 43% reduction in unprotected sex among the people who received HIV counseling and testing (HCT) [9].

In the mid-1980s the Ugandan government launched a “Zero Grazing” public health campaign meant to educate people about how HIV spreads, to encourage compassion for the afflicted and pragmatic behavior change, especially sexual faithfulness [10]. The campaign was linked to a broad-based social movement that brought HIV into the open and helped break up the networks that were spreading the virus-one of the few country-oriented success stories in the world to date [6, 10]. In the end, there was one clear winner: Uganda [10]. From 1992 to 2003, the HIV rate in Uganda fell by two-thirds, Dr. Epstein writes, “a success that saved perhaps a million lives”. Dr. Epstein makes a good case for the efficacy of a pervasive grass-roots effort, with ordinary people talking openly about AIDS and caring for the sick and orphaned in hundreds of small community initiatives in a “spirit of collective action and mutual aid” [10].

The wave of new as well as old infection in Uganda has shifted to older age groups [11, 12] with both HIV incidence and prevalence in Uganda's mature HIV epidemic having stopped declining around 2000 and hence remaining more or less stable [11, 12, 13]. Women, urban dwellers and residences of the post-conflict Gulu, northern Uganda are more disproportionately affected [11, 14]. There is evidence to suggest that despite a significant decline in HIV prevalence between 1992 and 2002, HIV prevalence has stagnated over the last 5–9 years at between 6.1 and 6.5% and may be rising in some parts of the country or specific population groups [11–15].

Worse still, evidence from program reports and research studies have revealed deterioration in most behavioral indicators amidst reports of complacency for HIV prevention at individual and organizational levels [6, 15, 16]. Researches in this area of HCT have long indicated that providing people with comprehensive information on reducing HIV risk is most effective at preventing new infections [17, 18]. Nevertheless, Uganda is one of the most successful countries in Africa in reducing the prevalence of HIV/AIDS significantly from 18% (1992) to 6.4% (2005) over the last two decades [2, 9, 11, 19, 20]. The 2004/05 Uganda Ministry of Health National HIV Sero and Behavioural Survey (NHSBS) established that for both sexes, HIV prevalence is highest among those aged 30–34 and lowest in the 15–19 years age category [2, 6, 9, 11, 18]. This represents an upward shift in age of highest prevalence over the last two decades [6, 9]. In addition, women have higher prevalence of HIV across all age categories and regions of the country (7.3% in women compared to 5.2% in men aged 15–59 years) [11]. The normalization of HIV/AIDS by some sections of communities such as in Gulu, Northern Uganda has also contributed to some form of disengagement from preventive behaviors including HCT [21, 22]. The major objective of this study was to find out the knowledge, misconceptions, attitude and practices of post-conflict young adults of Gulu, Pece division on HIV counseling and testing (HCT).

## Methods

### Study population and sample

We conducted questionnaire guided in-depth interviews with the young adults of Commercial Road Parish, one of the parishes in Pece Division of Gulu Municipality in Northern Uganda from January to June 2010. Gulu town is a regional city and commercial centre which is strategically located and endowed with its transport terminals and pivoted role in the vast and profitable distribution of goods in the region. Many people were displaced into camps famously known as the internally displaced peoples camps (IDPS) for safety from the 20 year old insurgency. According to the Revised Gulu District Development Plan 2009/2010, Pece division has a population of 36,133 and composed of 4 parishes and 16 villages. One of these parishes is Commercial Road Parish which was chosen as a study site purposively because of its high population density and large number of youths. This parish was massively affected by this displacement of persons from the neighboring rural communities. A prospective study was conducted among the young adults 15 to 35 years using a prepared questionnaire designed for data collection. A total sample of 86 respondents was selected, interviewed from different households to complete the numbers for the study. The study variables were controlled by interviewing only respondents, who were residents of the parish, consented and of qualifying age.

### Questionnaire administration

Questionnaires were administered by the interviewers at the respondents’ residence to collect the data required. Informed verbal and written consents were obtained from the respondents before the in-depth interviews and they had to freely agree to join the study and provide the required information. The questionnaire was pilot tested among the young adults of Layibi Division, Cereleno Parish in Gulu Municipality. This parish was selected because of its similar demographic characteristics with the population in Pece Division. The Questionnaire was designed to collect the background demographic data and the detailed information on knowledge, misconceptions, attitude and practices on HIV counseling and testing (HCT) among these respondents. After the pilot test, it was improved to help the respondents recall the HCT events and space added to consider other relevant information. The tested questionnaires were then administered to the respondents individually to obtain the accurate information needed. In order to avoid an unnecessary semantic misunderstanding, the questionnaire was written in simple English and translated into Luo, the main regional language by the investigators in conjunction with a trained interviewers and interpreters.

The interviewers were mainly medical students and some of whom Luo was not their first language and this therefore formed part of the limitation of the study. Respondents were asked open-ended questions to describe their knowledge (scaled as inadequate to adequate), attitudes (using graphic rating scale, Brand 1) and practices on HCT and also made to restate their knowledge about HCT before entry into the questionnaire. HIV counseling and testing (HCT) was defined as the process by which an individual, couple, or family receives HIV testing and counseling on HIV prevention, treatment, care, and support. There were extra spaces on the questionnaire that allowed space for providing qualifying remarks; these aided considerably in giving answers to specific questions and providing additional information which assisted the researchers.

Ethical clearance and approval of the study was obtained from the research committee and the administration of Gulu Regional Referral Hospital. During the data collection process, confidentiality, respect, privacy and high moral principles were observed. In the households the respondents were sampled for the interviews based on the village, informed consent and age. A maximum of 2 respondents were sampled per household. Overall 60 households were selected and a sample of 86 respondents was obtained. The data obtained were put in descriptive form and analyzed in percentages and fractions.

## Results

### Ages

The respondents’ ages ranged from 15 to 35 with a mean of 31 years; majority of the participants (N = 43) 50% were between the age of 31–35 years (Table 1).

**Table 1:** The Socio-economic characteristics of respondents

**The ages of the respondents**	**Frequency**	**Percentage**
15–19	9	10.47
20–24	21	24.42
25–29	13	15.12
30–35	43	50.00
Total	86	100.00
**Level of Education of respondents**	**Frequency**	**Percentage**
Primary	14	16.28
Secondary	34	39.53
Tertiary	38	44.19
Total	86	100
**Marital Status of the respondents**	**Frequency**	**Percentage**
Married	32	37.21
Not Married	52	60.47
Divorced	2	2.33
Total	86	100.00
**Where the respondents live**	**Frequency**	**Percentage**
With Parents	83	96.51
On their own	3	3.49
Total	86	100.00
**Distances of the respondents to the HCT centers**	**Frequency**	**Percentage**
<1Km	80	93.02
1 Km	5	5.81
> 1 Km	1	1.16
Total	86	100.00
**Occupation of the respondents**	**Frequency**	**Percentage**
Students	4	4.65
Pupils	3	3.49
Family Business	30	34.88
Peasant farmer	49	56.98
Total	86	100.00

### Gender

Female to male ratio of 1.1:1 (53%:47%).

### Marital status

Only 37.2% of the respondents were married, 60.5% were never married and 2.3% were divorced or separated (Table 1).

### Knowledge about HCT

Most respondents (93%) had adequate knowledge about HCT while 7% had inadequate knowledge (Table 2).

**Table 2:** Knowledge, attitude and practices of the respondents to HCT

**(a) Respondents' Knowledge on HCT**		
**Respondents’ knowledge**	**Frequency**	**Percentage**
Adequate Knowledge	80	93.02
Inadequate Knowledge	6	6.98
**Knowledge on the benefits of HCT**	**Frequency**	**Percentage**
Knew the benefits of HCT	84	97.67
Did not know any benefit of HCT	2	2.33
**Sources of HCT information to the respondents**	Frequency	Percentage
Health workers	36	41.86
Friends	11	12.79
Radios	23	26.74
Others e.g. Newspapers	16	18.60
**(b) Respondents' Practices to HCT**	Frequency	Percentage
Attendance of HCT by respondents		
Attended HCT	31	36.05
Has not attended HCT	55	63.95
**Number of respondents that attended HCT as couples**	Frequency	Percentage
Attending HCT with partner	17	19.77
Attending HCT without a partner	69	80.23
**Recommendation on who should undertake HCT**	Frequency	Percentage
Students	34	39.53
Barmaids	33	38.37
Married couples	10	11.63
Others	9	10.47
**The number of sexual partners of the respondents**	Frequency	Percentage
single partner	19	22.09
Two partners	28	32.56
More than two partners	39	45.35
**The Gender of the respondent with a single sexual partner**	Frequency	Percentage
Male	36	41.86
Female	50	58.14
**(c) Attitudes of the respondents to HCT**		
Respondents attitudes towards many people undertaking HCT	Frequency	Percentage
Feel Happy	72	83.72
Not bothered by others' action	10	11.63
Could only lament	2	2.33
**Person to whom respondents could openly disclose their status**	Frequency	Percentage
Health workers	60	69.77
Friends who keeps secrets	7	8.14
Wife/Husbands	5	5.81
Boy/Girlfriends	4	4.65
**The Number of HCT sessions attended by respondents**	Frequency	Percentage
Visited once	3	9.68
Visited Twice	18	58.06
Visited more than twice	10	32.26
**Attendance of HCT by Gender**	Frequency	Percentage
Male	9	29.03
Female	22	70.97

### Sources of HCT information

Most respondents (41.3%) received HCT information from health workers, 12.6% from friends, 26.9% from the media (mainly FM Radio) and 19.2% from others e.g. Newspapers, posters, social functions and schools (Table 2).

### Knowledge about benefits of HCT

Most respondents mentioned more than 2 benefits of HCT while 2.3% could not mention any benefit but recognized the danger of contracting HIV/AIDS (Table 2).

### Number of sexual partners

Few respondents (22.1%) had a single sexual partner, 32.5% had two sexual partners and 45.4% had more than three sexual partners simultaneously. Of those with a single sexual partner, (58%) were females and (42%) males (Table 2).

### Opinion of respondents on who should undertake HCT

Most respondents (39.5%) recommended that HCT services should be undertaken by students, 38.4% Barmaids, and 11.6% married couples and 10.4% to the others (Table 2).

### Attitudes of respondents towards open disclosure of HCT

Most respondents (88.4%) expressed that it was proper to discuss HCT results openly to colleagues, friends and the public. However 11.6% of the respondents reported that it was not proper to have open disclosure of HIV status. Those who expressed positive attitudes towards open disclosure of HCT results reported that they would feel free to talk about it mainly with a health worker (70%) or a friend who keeps secrets (8%), a wife/husband (6%) or a girl/boyfriend (4.4%). However, those who said it was not proper to discuss HCT openly said they would only consider discussing it with a health worker otherwise they would prefer to keep the HCT information secret to themselves (Table 2).

### Attitudes of respondents towards those undertaking HCT

Most respondents (84.9%) felt happy about more people undertaking HCT while 12.8% were not interested about others and 2.3% could only sympathize (they felt they could not do much about their decision) with those that were not undertaking HCT (Table 2).

### Attendance of HCT among respondents

Only 36.1% respondents had undertaken HCT and 63.9% had not undertaken it and all were not planning to do so (Table 2).

### Distribution of attendance of HCT by Gender

Most respondents (70.9%) who attended HCT were females and only 29.1% were males (Table 2).

### The number of HCT sessions attended

Only (36.1%) respondents attended HCT with 9.7% having visited HCT once, 58.1% visited twice and 32.2% more than twice. The number of times HCT is undertaken is part of the protocol for HCT. Three visits are recommended standard for HCT in Uganda in order to confirm diagnosis and follow up on post test services (Table 2).

### Attending HCT as a couple

Out of the 36.1% that undertook HCT, 80.6% went for HCT without their partners and 19.4% attended with their partners. Among those who did not undertake HCT with their partners, 68.0% discussed test results with their partners and 32% of those who tested as a couple did not disclose status to their partner (Table 2).

### Reasons for nondisclosure of HCT results

Most respondents (70%) did not disclose because of fear of stigmatization, 8% for fear of rejection, 7% for fear of continuous denial, 10% for fear of knowing status and implications, 5% for fear of losing self-esteem (Table 3).

**Table 3:** Reasons for non-disclosure and ratings for knowledge and attitudes

**(a)Reasons for non-disclosure of HIV status among respondents**	**Frequency**	**Percentage**
Fear of stigmatization	17	68.00
Fear of Rejection	2	8.00
Fear of Knowing status	2	8.00
Fear of Denial	3	12.00
Fear of Loss of self esteem	1	4.00
Total	25	100.00
**(b)Knowledge Rating of respondents to HCT**		
	**Ratings**	
**Variables**	**Adequate**	**Inadequate**
The meaning of HCT	Can define HCT	Cannot define HCT
How HCT is conducted	Can explain process of HCT	Cannot explain process of HCT
The Uses of HCT	Mentions 4 uses of HCT	Couldn't mention any of the Uses of HCT
Where HCT is conducted	Mentions 3 HCT sites	Knows no HCT sites
What are the obstacles to accessing HCT	Mentions at least 1 obstacle to HCT	Could not mention any obstacle to HCT
**(b)Rating of the Respondents' attitude to HCT**		
	**Ratings**	
**Variables**	**Poor**	**Good**
Views of respondents to open disclosure of HIV status	Negative	Positive
Undertaking HCT	Not willing to undertake HCT	Willing to undertake/Has undertaken HCT
Those who haven't taken HCT	Does not care	willing to convince others to undertake HCT
Knowledge of HCT sites	Not interested in knowing	Knows the HCT sites in the area

## Discussion

### Age

The majority of respondents were in the age range 31–35years. A national survey, UDHS (2006) showed that in the age group of 15–49 years; 28.3% females and 35.8% males have comprehensive knowledge about HIV/AIDS while for the ages 15–24 years; 29.5% women and 35.3% men do have comprehensive knowledge [22]. The age group of 15 to 35 years was considered by the authors as the most vulnerable group in the spread of HIV basing on previous researches in the region.

### Gender

Most respondents were females. This reflects the normal population distribution of the people of Uganda [11] although in this particular study, the researchers noted that they more often found female youths at home and the likelihood for them being interviewed were increased. We wish to acknowledge that, this may be a limitation to this study. At this point, the authors wish to note that due to existing societal gender inequalities, women were often economically, culturally and socially disadvantaged as compared to the men because women lacked equal access to treatment, financial support and education on HIV/AIDS [23]. Cultural standards for female versus male sexual behaviours differ in Uganda and it is often believed that promiscuous woman deserved to become infected, while promiscuous men were merely proving their manhood [23]. Gender power dynamics assert themselves in sexual partnership and this may explain partly the reason for the high prevalence of HIV/AIDS among the women in Uganda [6].

### Level of Education

Most respondents had attained education beyond primary school while a few had only attained primary school education. This adequate level of knowledge of respondents to HCT we speculate was more likely to have been due to their good educational standard. Their sources of information were mainly from health workers, radios, friends and others such as newspapers. It should be noted here that English is not the first language for all the respondents and English language is mostly learnt in schools. The medium of communication of HCT to respondents were mainly made in English. The few who had inadequate knowledge of HCT and could not define HCT were mainly those who had only attained primary school education. There is a good media coverage (several FM radio stations in the area) in which health workers regularly disseminated information about HCT to the youths for example on behaviour change messages, HCT, and the sites where testing take place. It is true therefore that education plays a major role in understanding and awareness of the environment [23]. Education and communication are tools that reduce stigma from communities in order to reduce the escalation of HIV/AIDS [23]. Ignorance is always a barrier to advancing of any knowledge and studies have shown that when people have been prepared through the right education, they will be more willing to get tested, to seek treatment and to change their behaviours [23, 24]. They will be less affected by stigma, shame and guilt and will be able to contribute to an equally tolerant society [23–25].

Uganda has a plethora of IEC/BCC campaigns for cross-sections of the population using mass media and interpersonal communications (IPCs) [22]. A number of sex education programmes even for young people that are outside the school system are in place including young empowered and healthy (YEAH) and outreach and training program [22]. Overall, awareness about HIV/AIDS and correct knowledge about modes of transmission and, therefore, ways of prevention is significant [21, 22].

### Marital status

Most respondents were not married (single) and the males claimed that they did not have the resources to complete their marriages and to live on their own. In many sub-Saharan African countries, a large proportion of HIV transmission occurs within married relationships or similar unions [24]. Counseling and testing is particularly important for pregnant couples, but male partner involvement in antenatal clinics (ANCs) is usually low [24, 26–29]. Studies from Nairobi and Lusaka indicated that only 5%–9% of women came with their partners for VCT in antenatal clinics, despite community outreach [25]. Similar findings were reported in Uganda as too [6].

### Sexual activity

Most respondents had two or more sexual partners simultaneously while only a quarter had a single partner in the last five years. Many youths in Uganda feel that they are invincible and that nothing bad will happen to them, many more still belief that HIV does not cause AIDS [23, 27, 29]. Only a third of the respondents had ever undertaken HCT. This correlates with the findings and explanation from HIV/AIDS sexual behaviour and intravenous drug use [30] that individuals who are more concerned about their health may be more likely to seek testing and counseling for HIV or undergo diagnostic evaluation more frequently than those who are less concerned about their health [30]. Multiple concurrent partnerships, in which consistent condom use tends to be low, combined with low levels of male circumcision, are the key drivers of HIV/AIDS in Southern Africa [31–33].

Only 19 respondents in our study had a single sexual partner in the last five years. At any given time, a significant percentage of men are engaging in multiple sexual partnerships with women – a situation that may facilitate the spread of sexually transmitted infections, including HIV/AIDS [34]. This we suspect could be explained in terms of cultural orientation of the people in this region in which males have the liberty to have as many wives as possible (polygamous community). In terms of sexual behavior, the UHSBS report showed that youths who were orphans or vulnerable children were slightly more likely to have sex by age 15 than other youth and young women classified as orphans and vulnerable children (OVC)[6]. Various factors associated with social and cultural values, beliefs, perceptions and practices are known to influence dominant sexual behaviours that have a bearing on HIV prevention [6, 34]. There is increasing evidence to show that the number of multiple sexual relationships increased between 2001 and 2005 from 25% to 29% in men and from 2% to 4% in women in Uganda [34]. Among married couples, the proportion reporting extra-marital sex during the same period increased from 14% to 29% among men but remained stable at 3% among women [21, 34, 35]. Historically, the zero grazing campaigns of the late 1980s had a great impact on HIV transmission contributing to the decline in prevalence from 18% to 6.4% [34]. Married and co-habiting couples are thus a key population group that needs to be targeted with an HIV prevention package specifically designed to suite the uniqueness of marital relationships [34, 35].

### Respondents’ recommendation on who should undertake HCT

The majority of respondents recommended students, barmaids, married couples and to others e.g. teachers, civil servants and motorcyclists. The respondents reported that students (usually 15years and above) were sexually active and that they have sex with barmaids who mainly engage in commercial sexual activity. For many years, many societies have associated HIV/AIDS with homosexuality, injection drug use, promiscuity and prostitution and as a consequence the disease has been perceived by religious groups as punishment for the moral failings, thus inflicting guilt on those infected or affected [23].

### Attitudes of respondents to HCT

Most respondents had a positive attitude to HCT. They approved and encouraged others to undertake it. The few who did not approve it had one simple answer, “even if you tested and were found HIV positive, what would you do other than wait to die”. Most respondents were willing to discuss HCT openly although a few believed it was improper to discuss it openly except with health workers, close friends, husbands/wives and girl/boyfriends due to fear of stigmatization. Stigma impedes the fight against HIV/AIDS and causes reluctance to disclosure, thus promoting secrecy, finally arguably abetting transmission [23]. Stigma drives the epidemic underground leaving health seeking and preventive measures minimally utilized [23]. In a community where stigma prevails, no open discussions about HIV/AIDS take place and this among other things, prevents identification of AIDS orphans and promotes the oppression of women [23].

In this study, only a few respondents had undertaken HCT. In a survey of males and females of ages 14 to 21 years, about 90 percent of 210 Ugandans and 75 percent of 122 Kenyans who said they had not received VCT services reported that they wanted to be tested [35, 36]. However, in these studies, some young people feared testing, some worried that their test results would be positive [35, 36]. Others were concerned that their test results would not remain confidential, that they might lose their partners, and that the services would be costly or be provided in an inconvenient locations [21].

Most respondents who undertook HCT were females. Females were more willing to undertake HCT than males in Uganda [36]. Females cared much about their individual health compared to males and more so during the antenatal visits they get tested for HIV as part of the procedural requirements unless any specific objection is raised [36, 37]. In a Ugandan study of 2002, three hundred sixty nine (369) young people ages 14 to 21 years who had sought VCT, young women who decided to get tested tended to do so if they were about to be married, enjoyed their partners′ support, and knew their partners and were willing to pay for the services [37]. Nearly two of every three girls said their partners encouraged them to be tested. In contrast, boys were more likely to decide on their own to be tested and to pay for testing themselves [37]. A third of boys said their decision to seek VCT testing was influenced by partners; a third, by friends; and another third, by no one [37].

Studies in Uganda and elsewhere have shown that people who know their HIV status are more likely to protect themselves and others from HIV infection [39]. However, over 75% of adults in Uganda do not know their HIV sero-status [34]. Most respondents in this study attended HCT without their sexual partners and disclosed their results only when they were HIV negative. Many people live in denial, or fail to disclose their HIV/AIDS status in order to protect their families from social condemnation [23, 27, 39, 40]. In a previous study conducted in this region, the reasons for nondisclosure were obtained from 20 participants and the most commonly cited reasons for nondisclosure included need for privacy, fear of rejection, and fear of physical abuse [36, 41].

In these expanded efforts to provide HCT services to young people, key programmatic challenges are confidentiality, parental consent, adequate counseling, and ongoing support [41]. Unless VCT is strictly confidential, young people (especially women) run the risk–as do adults–of being stigmatized, suffering violence, and being disowned by family members or partners [36, 41]. One of the key challenges for HCT programs in Uganda has been deciding whether to involve a youth′s parents in the VCT process, gaining approval for testing and reporting of results [36]. Ideally, each country would determine informed consent procedures for using VCT [36, 38, 42]. In Kenya, the national VCT guidelines issued in 2001 advised that “mature minors” do not need parental consent. “Mature minors” include those individuals younger than 18 years who are “married, pregnant, parents, or those engaged in behavior that puts them at risk, or are child sex workers”[38]. A growing body of evidence suggests that making HIV testing part of the standard care reduces the stigma associated with the disease and increases the number of those choosing to be tested [43]. Routine testing, mass media campaigns promoting the value of knowing the HIV status and learning the benefits and wide availability of treatment, have dramatically increased the counseling and testing services in Botswana [43].

## Conclusion

There is adequate knowledge on most aspects of HCT by the young adults. There is good attitude but poor practice and misconceptions to HCT. The Gulu young adults should be supported in a special program to enable them undertake HCT and access other services for HIV/AIDS care and management.

## References

[1] The Voluntary HIV-1 counseling and testing efficacy group (2000). Efficacy of voluntary HIV-1 counseling and testing in individuals and couples in the Kenya, Tanzania, and Trinidad: A randomized Trial. Lancet.

[2] Serwadda David, Mugerwa Roy, Sewankambo Nelson (1985). Slim Disease: A New Disease in Uganda and its Association with HTLV-III Infection. Lancet.

[3] UNAIDS (2010). UNAIDS report on the global AIDS epidemic.

[4] Horizons Program (2001). HIV Voluntary Counseling and Testing among Youth: Results from an Exploratory Study in Nairobi, Kenya, and Kampala and Masaka, Uganda.

[5] Baggaley Rachel, Kayawe Ignatius, David Miller, Lamptey Peter R, Gayle Helene D (2001). Counseling, testing and Psychosocial support in HIV/AIDS prevention and care in resource constrained settings: A handbook for the design and management of programs.

[6] National Health sero behavioral survey (NHSBS) (2007). Uganda Ministry of Health HIV sero and behavioral survey: GoU/MoH/ACP report (2004/2005).

[7] Juma Milka, McCauley Anne, Kirumira Edward (2002). Gender variations in uptake of VCT services among youth in Uganda.

[8] UNAIDS (1999). Knowledge is Power: Voluntary HIV Counseling and Testing in Uganda. GoU/MoH/UNAIDS/ACP report.

[9] Uganda National Sero-Survey (UNSS) (2008). Uganda National sero-survey (UNSS of 2004/2005).

[10] Epstein Helen (2007). The invisible cure. Africa, the west and the fight against AIDS.

[11] United Nations Development Program (UNDP) (2008). Macro-economy HIV/AIDS assessment in Uganda. GoU/UNDP report.

[12] Shafer Ann, Biraro Samuel, Nakiyingi-Miiro (2008). HIV prevalence and Incidence are no longer falling in southwest Uganda: Evidence from rural population cohort 1989-2005. AIDS.

[13] Uganda Ministry of Health (MOH) (2009). HIV/AIDS epidemiological surveillance report, GoU/MoH/ACP.

[14] Uganda AIDS Commission (AIC) (2006). Accelerated HIV prevention, a roadmap towards universal access to HIV prevention in Uganda. GoU/MoH/ACP report.

[15] UNAIDS (2010). UNGASS country progress report: Uganda'. GoU/UNAIDS report.

[16] Opio Alex, Mishra Victor, Hong Ron (2008). Trends in HIV-related behaviours in Uganda, 1989–2005: Evidence of a shift towards more risk-taking behaviours. J Acquir Immune Defic Syndr.

[17] Okware Sam, Kaggwa Paul, Onyango Saul (2005). Revisiting the ABC Strategy: HIV prevention in Uganda in the era of antiretroviral therapy. Postgrad Med J.

[18] Uganda AIDS Commission (UAC) (2008). Report on Implementation of National HIV and AIDS Strategic Plan FY 2007/2008. GoU/MoH/UAC report.

[19] Uganda AIDS Commission (UAC) (2008). Uganda AIDS Commission. National Strategic Framework for HIV/AIDS' GoU/MoH/ACP report.

[20] Low-Beer Daniel (2002). HIV-1 incidence and prevalence trends in Uganda. Lancet.

[21] Uganda AIDS Commission (UAC) (2009). Report on Implementation of National HIV and AIDS Strategic Plan FY 2007/2008, 2011/2012. GoU/MoH/ACP report.

[22] Uganda Demographic Health Survey (UDHS) (2006). The Uganda demographic and Health survey. GoU/MoH/ACP report.

[23] Wambayi Edith (2005). External consultant in consultancy Africa intelligence's HIV/AIDS unit (hiv.aids@consultancyafrica.com).

[24] Becker Stanley, Gray Ron (2008). Source of new infections in generalized HIV epidemics. Lancet.

[25] Farquhar Carey, Kiarie James, Richardson Barbra (2004). Antenatal couples counseling increases uptake of interventions to prevent HIV-1transmission. J Acquir Immune Defic Syndr.

[26] Tumwesigye Elioda (2008). Knowledge of HIV status among Ugandans.

[27] Semrau Katherine, Kuhn Lindsey, Vwalika Chris (2005). Women in couples antenatal HIV counseling and testing are not more likely to report adverse social events. AIDS.

[28] Mugyenyi Peter (2008). Genocide by Denial.

[29] Mugyenyi Peter (2008). Testimony to US Congress: House Committee on Foreign Affairs, Subcommittee on Africa and Global Health.

[30] Charles Turner, Heather Miller, Lincoln Moses (1988). AIDS-sexual behaviour and intravenous drug use in New York City. Science.

[31] Mohapeloa Veronica (2006). Multiple sex partners driving HIV.

[32] Childs Dan and Williams Carla (2007). One in 10 men has multiple sex partners.

[33] UNAIDS (2005). Resource Needs for an expanded response to AIDS in low and middle-income countries. Presentation to the UNAIDS program board meeting.

[34] Wabwire-Mangen Fred, Odiit Martin, Kirungi Wilford (2008). Modes of Transmission Study, Analysis of HIV Prevention Response and Modes of HIV Transmission, the Uganda Country Synthesis Report, GoU/UNAIDS/UAC.

[35] WHO/UNAIDS/UNICEF (2009). Towards Universal Access: Scaling up priority HIV/AIDS interventions in the health sector, GoU/UNAIDS/UNICEF report.

[36] Namwebya Jane, Turyagyenda Jackson, Baryarama Fulgentius (2000). Discordance rates among Ugandan couples seeking VCT services before having sex together.

[37] Menzies Nick, Abang Betty, Nuwaha Fred (2009). The costs and effectiveness of four HIV counseling and testing strategies in Uganda. AIDS.

[38] NASCOP (2001). Kenya Ministry of Health, National AIDS and STD Control Programme. National Guidelines for Voluntary Counseling and Testing.

[39] UNICEF/UAC (2003). The District Response Initiative on HIV/AIDS in Uganda-Action Research: National Synthesis Report.

[40] Kalichman Seth, Simbayi Leickness (2003). HIV testing attitudes, AIDS stigma, and voluntary HIV counseling and testing in the black township in Cape Town, South Africa. Sex Transm infect.

[41] Kirungi Wilford, Musinguzi James, Madraa Elizabeth (2006). Trends in antenatal HIV prevalence in urban Uganda associated with uptake of preventive sexual behaviour. Sex Transm infect.

[42] Kirungi Wilford (2008). Review of HIV Prevention Activities in Uganda, Modes of Transmission Study: Uganda.

[43] Morris and Ferguson (2006). Estimation of the sexual transmission of HIV in Kenya and Uganda on the trans-Africa highway: the continuing role for prevention in high risk groups. Sex Transm Infect.

